# Treatment of COVID-19 by stage: any space left for mesenchymal stem cell therapy?

**DOI:** 10.2217/rme-2020-0189

**Published:** 2021-05-14

**Authors:** Gaia Spinetti, Elisa Avolio, Paolo Madeddu

**Affiliations:** ^1^IRCCS MultiMedica, Milan 20138, Italy; ^2^Bristol Medical School, Translational Health Sciences, University of Bristol, Bristol BS2 8HW, UK

**Keywords:** acute respiratory distress syndrome, coronavirus, cytokine storm, epithelial cells, infection, mesenchymal stem cells, vascular cells

## Abstract

In many countries, COVID-19 now accounts for more deaths per year than car accidents and even the deadliest wars. Combating the viral pandemics requires a coordinated effort to develop therapeutic protocols adaptable to the disease severity. In this review article, we summarize a graded approach aiming to shield cells from SARS-CoV-2 entry and infection, inhibit excess inflammation and evasion of the immune response, and ultimately prevent systemic organ failure. Moreover, we focus on mesenchymal stem cell therapy, which has shown safety and efficacy as a treatment of inflammatory and immune diseases. The cell therapy approach is now repurposed in patients with severe COVID-19. Numerous trials of mesenchymal stem cell therapy are ongoing, especially in China and the USA. Leader companies in cell therapy have also started controlled trials utilizing their quality assessed cell products. Results are too premature to reach definitive conclusions.

## Epidemiology & emergence of new variants

In late 2019, infection with a novel beta-coronavirus, subsequently termed SARS-CoV-2, was reported in Wuhan, China, where live animals were sold. Since then, the rapid spread of the virus has led to a global pandemic of COVID-19. As of 3 February 2021, over 103 million cases of COVID-19 (in accordance with the applied case definitions and testing strategies in the affected countries) have been reported, including 2.24 million deaths. Severe lung disease, characterized by ‘acute respiratory distress syndrome’ (ARDS), and multi-organ dysfunction with disseminated intravascular coagulation represent the most severe complications [1]. Myocardial injury is present in more than a quarter of critical cases, manifesting either acutely on presentation or more insidiously as illness severity intensifies [2–4]. Moreover, COVID-19 shows a strong age gradient in the risk of death [5]. The long-term health consequences of COVID-19 remain largely unclear. A recent cohort study of 1733 patients with confirmed COVID-19, who had been discharged from Jin Yin-tan Hospital (Wuhan, China) between 7 Jan and 29 May 2020, showed the frequent persistence of fatigue or muscle weakness, sleep difficulties and anxiety or depression. Patients with more severe disease during hospitalization had functional and radiographic evidence of pulmonary alterations [6].

Multiple variants of the virus that cause COVID-19 are circulating globally. The UK B.1.1.7 variant appeared in the fall of 2020, spreading more easily and quickly than other variants [7]. In January 2021, preliminary evidence was provided that this variant may be also associated with an increased risk of death, but more studies are needed to confirm this finding [8]. The South African 501Y.V2 variant emerged in Nelson Mandela Bay metropolitan area in early October 2020, then spread quickly to become the predominant virus lineage in the Eastern and Western Cape Provinces by the end of November 2020. Cases caused by this variant were reported in the USA and United Kingdom at the end of January 2021 [9]. In early January 2021, a variant called P.1 was discovered during routine screenings at an airport in Japan on passengers from Brazil [10]. This variant contains a set of additional mutations that may compromise the recognition by antibodies (Abs).

## Clinical presentation

COVID-19 can present a variety of manifestations ranging from asymptomatic infection to critical disease (reviewed in [11–13]).

Although no precise guidelines exist to define grade severity, mild disease is diagnosed as a condition characterized by fever, cough, sore throat and myalgia. Some patients have gastrointestinal symptoms including nausea and diarrhea [14]. Anosmia and ageusia were initially not considered but were then realized to manifest in >60% of patients, being more frequent in women [15].

Patients with moderate disease may suffer shortness of breath, presenting 5–8 days after initial symptom onset, as the first indication of a worsening state [16]. Chest radiography confirms lower respiratory tract disease but with a blood oxygen saturation of ≥94% while the patient is breathing ambient air.

Severe disease is characterized by tachypnea, oxygen saturation ≤93% and radiographic evidence of lung infiltrates in >50% of the lung field involved within 24–48 h [17].

The definition criteria for critical COVID-19 include respiratory rate ≤30-times/min, pulse oxygen saturation at rest ≤93%, the partial pressure of PaO_2_/FiO_2_ ≤ 300 mmHg, a requirement for mechanical ventilation and shock [18].

ARDS is the main cause of poor prognosis in critically ill patients [19], manifesting in 42% of those with pneumonia, and 61–81% of those requiring intensive care [20]. Classically, ARDS is defined by the following criteria: acute hypoxemic respiratory failure; presentation within 1 week of worsening respiratory symptoms; bilateral airspace disease on chest x-ray, computed tomography or ultrasound that is not fully explained by effusions, lobar or lung collapse, or nodules; and cardiac failure [21]. Anatomically, there is a damage to the pulmonary capillary endothelium and alveolar epithelium, leading to inflammatory exudate in the alveoli and pulmonary edema. An important feature of COVID-19-associated ARDS is the occurrence of increased thrombotic and microvascular complications. Several studies have assessed the circulating and alveolar levels of markers associated with endothelial damage and platelet activation in critically and noncritically ill patients [22–24]. High levels of fibrin degradation products, D-dimer, soluble thrombomodulin, von Willebrand factor and PAI-1, and decreased CRP were found in critical patients compared with noncritical patients [25]. Mortality was significantly correlated with von Willebrand factor and soluble thrombomodulin among all patients, while high soluble thrombomodulin concentrations were associated with lower rates of hospital discharge and a lower likelihood of survival [25]. These data support the view that the early identification and treatment of endotheliopathy could improve outcomes in patients with COVID-19.

**Figure 1.**
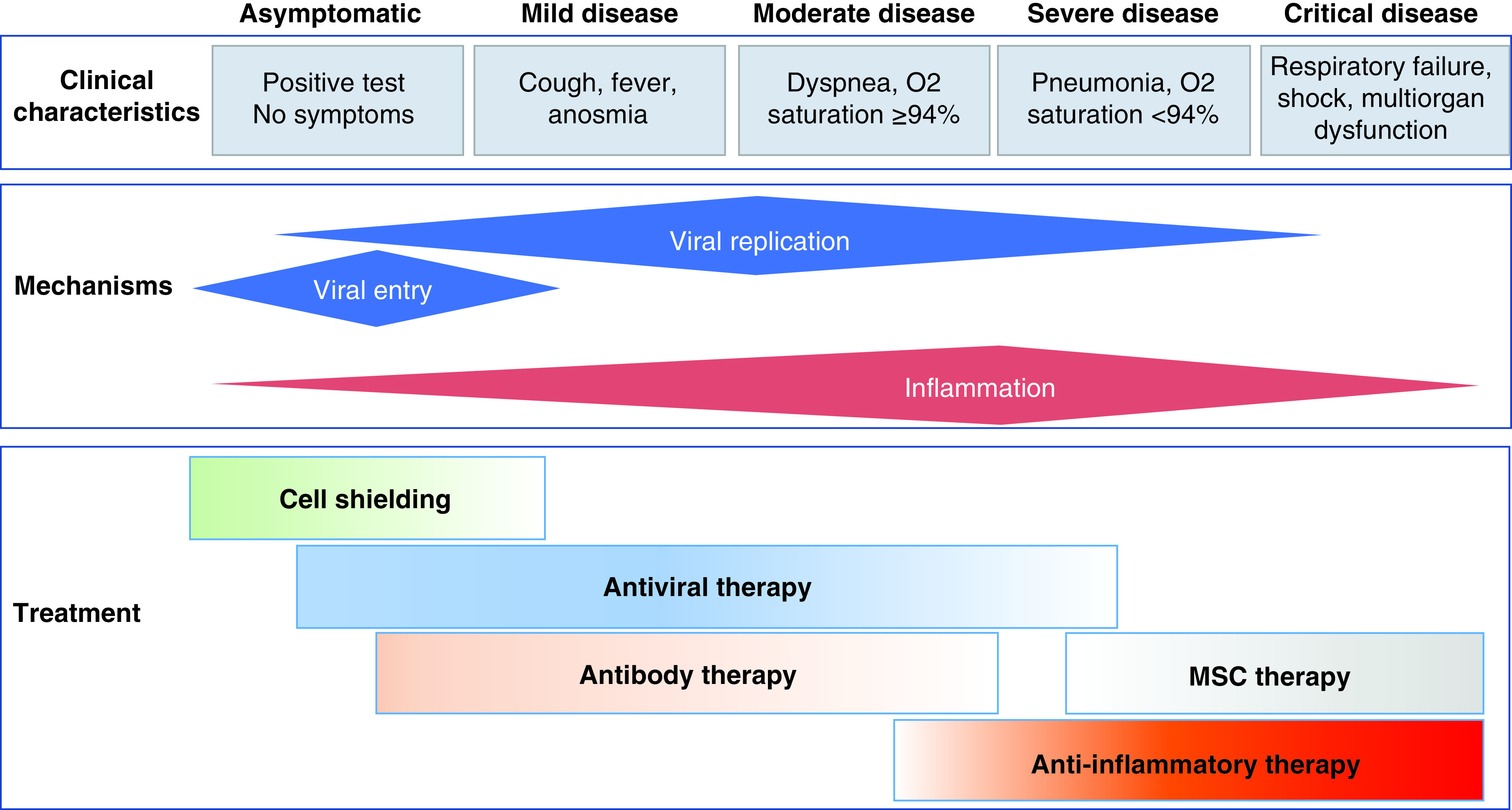
Treatment protocol according to the disease stage and target. The upper panel identifies clinical signs of disease progression. The middle panel shows the virus-intrinsic and host-related mechanisms. The lower panel shows the graded approach.

Altogether these clinical pieces of evidence support the adoption of graded, combinatory therapeutic protocols to control the evolution of symptoms [26]. In the next sections, we summarize the key pathogenic mechanisms providing a rationale for targeted therapies (Figure 1). We also focus on the rationale for using mesenchymal stem cell (MSC) therapy, illustrating the current landscape of ongoing clinical trials.

### Mechanisms of infection

Coronavirus particles consist of a helical structure, formed by the association between nucleocapsid (N) phosphoproteins and the viral genomic RNA, which is surrounded by a lipid bilayer where three or four types of structural proteins are inserted: the spike (S), the membrane (M), the envelope (E) proteins and, for some coronaviruses only, the HE protein [27]. While the M and E proteins are involved in viral assembly, the S protein is the leading mediator of viral entry following processing by host cell proteases, such as the serine protease TMPRSS2 [28], and subsequent binding to cellular receptors [29]. SARS-CoV-2 receptors are classified as entry receptors, some being expressed by different host cells but others exclusively by a specific cell population, and attachment receptors.

SARS-CoV-2 infects the respiratory tract by adhering to components of the airway’s epithelia, namely the carbohydrate chains of proteoglycans and glycosphingolipids, and then engaging with often multiple, cellular entry receptors [30]. By disrupting the multilayered pulmonary barrier formed by epithelial cells, endothelial cells and pericytes, SARS-CoV-2 can spread through the circulation and infect/damage other organs including the heart [31,32].

## Anchor receptors

The first target for therapy of COVID-19 is shielding epithelial and endothelial cells from the virus contact. The SARS-CoV-2 anchor receptor HSPG, which is ubiquitously expressed within the proteoglycan-rich glycocalyx layer on the cell surface, allows the virus to make primary contact with permissive cells [33].

An association between the HSPG syndecan-1 and the CD147 entry receptor is reportedly essential for cyclophilin B-induced activation of p44/42 MAPKs and promotion of cell adhesion and chemotaxis [34]. Evidence indicates that there are heparin-binding sites both within and outside of the receptor-binding domain (RBD) on S-protein [35,36]. Heparan sulfate-binding sites are thought to reduce the specificity of the receptor required for cell entry. Moreover, attachment of SARS-CoV-2 to HSPG may stabilize the open conformation of the S protein, thereby promoting binding to the ACE2, which can only occur in the open conformation [36,37].

### Inhibition of the virus anchoring

Medications directed at HSPG may be valuable as a potential treatment for COVID-19 [38]. Heparin has recently been shown to block SARS-CoV-2 infection of permissible cells by inducing conformational changes of the S protein and competing with HSPG anchors [39–44]. Heparin is an anticoagulant that leads to bleeding risk in patients [45]. Therefore, researchers are focusing on safer HSPG competitors.

Lactoferrin is an 80-kDa iron-binding glycoprotein of the transferrin family, found in secretions such as milk, sputum, lung surfactant, and present in neutrophil granules. It is used for treating stomach and intestinal ulcers and diarrhea and has broad antiviral properties [46–49]. Interestingly, lactoferrin has been shown to protect against SARS-CoV-2 infection by blocking the S protein heparin-binding domains to a similar degree as heparin, without showing the anticoagulant adverse effects [50]. *In vitro* studies, showed that lactoferrin can inhibit viral infection in the early stages and is effective against SARS-CoV-2 in the post-infection phase. Furthermore, lactoferrin possesses immunomodulatory and anti-inflammatory effects [51] Considering the protein is available as a supplement, this would be an easy option to administer within the community and may be specifically suited to those in care homes. A recent trial of oral and intranasal administration of lactoferrin was conducted in 32 COVID-19 patients with mild to moderate symptoms [52]. The goal was to test the efficacy in improving symptoms and eliminating the virus. A dose of 1 g of liposomal apo-lactoferrin in ten capsules per day was administered orally for 30 days, in addition to the same form administered nasally three-times per day. All patients showed improvement in symptoms except fatigue, which continued in about a third of the group. Of note, the authors reported a decrease in D-dimer concentration, which is a biomarker of the severe outcome.

## Conventional & unconventional entry receptors

The widely expressed SARS-CoV-2 receptor, ACE2, belongs to a family of dipeptidyl carboxydipeptidases and has considerable homology to ACE [53]. Both enzymes are located on the plasma membranes of various cell types, including epithelial cells of the lung, vascular endothelial cells and pericytes, and their balance is essential to the maintenance of epithelial-vascular homeostasis [54,55].

ACE generates the vasoconstrictor and profibrotic Ang II, and degrades vasodilator BK into the pro-inflammatory metabolite [des-Arg^9^] BK. Conversely, ACE2 cleaves Ang II into the vasodilator Ang 1–7 peptide and degrades the ACE-generated [des-Arg^9^] BK peptide. The binding of SARS-CoV-2 S protein to ACE2 is detrimental in two ways, by allowing the virus to enter the cell and by causing ACE2 internalization/degradation, leaving the ACE-related peptide pathway unopposed and [des-Arg9] BK undegraded. The ensuing damage of the pulmonary epithelial-vascular barrier manifests in the form of vascular extravasation, lung congestion and typical acute respiratory syndrome as well as the systemic spread of the virus. Therefore, ACE2 has been considered a pivotal target for treatment.

Basigin/CD147, a plasma membrane protein associated with oligomannosidic glycans, has emerged as a novel receptor for SARS-CoV-2 [56]. It was found initially to be expressed in both lung epithelium and in immune cells [57], although, interestingly, in CD147-transfected HEK 293 cells, the S protein was not shown to bind [58]. The binding of S protein to CD147 may necessitate the recruitment of coreceptors present in the cells that constitutively express CD147. The best characterized binding partners of CD147 are monocarboxylate transporters (a family of molecules involved in lactate, pyruvate and ketone flux across the plasma membrane), caveolin-1, CD98, β1 integrin and CD44 (a major receptor for hyaluronan) [59].

### Inhibition of the virus entry receptors, processing protease & downstream mechanisms

Recombinant ACE2 has been used as a virus interceptor to reduce the SARS-CoV-2 infection in cardiovascular organoids [60] and is now investigated in a pilot trial as a treatment for patients with severe COVID-19 (ClinicalTrials.gov no.: NCT04287686). ACE2-blocking Abs have been also proposed as a treatment [61]. Nonetheless, ACE2-based approaches could be insufficient because of the ability of SARS-CoV-2 to use alternative pathways to enter human cells.

An open-label, clinical trial of meplazumab – a humanized monoclonal Ab against the CD147 receptor – showed clinical improvements in COVID-19 patients [62]. Potent individual Abs that simultaneously bind the RBD of the S protein provide an ideal solution to decrease the potential for virus escape mutants arising due to the selective pressure from a single-Ab administration or vaccination [63,64].

Combinatory blocking strategies may be considered, after the assessment of efficacy with a single blockade. Weinreich *et al.* reported the interim results of a trial in patients with early infection, combining two monoclonal Abs, casirivimab and imdevimab (together called REGN-COV2), raised against the S protein [65]. The patients were randomly assigned in a 1:1:1 ratio to receive a single intravenous infusion of either 2.4 g or 8 g of REGN-COV2 or placebo. In the first 275 patients, those who received either dose of REGN-COV2 had lower SARS-CoV-2 RNA levels than those who received placebo. A small number of patients (12) required a medically attended visit within the 29-day follow-up period, with a larger percentage in the placebo group. The US FDA has issued an emergency use authorization for REGN-COV2 to be administered for the treatment of mild to moderate COVID-19 in adults and pediatric patients.

Another trial conducted by Chen *et al.* [65,66] evaluated three doses (700, 2800 and 7000 mg) of a single monoclonal Ab, bamlanivimab (LY-CoV555), which was administered to 452 outpatients. Bamlanivimab was associated with a greater reduction in symptoms of COVID-19 than was placebo. An extension of the clinical trial is enrolling patients who will receive a combination of bamlanivimab and etesevimab (LY3832479) to overcome or prevent Ab resistance (ClinicalTrials.gov no.: NCT04427501).

Broadly neutralizing Abs represent an attractive opportunity for therapeutic drug stockpiling to prevent or mitigate future outbreaks of SARS-CoVs. An important study used a directed evolution approach to engineer three SARS-CoV-2 Abs for enhanced neutralization breadth and potency. The variant ADG-2 showed a strong binding activity to a large panel of sarbecovirus RBDs and neutralized representative epidemic sarbecoviruses with high potency [67]. Operation Warp Speed and the National Institutes of Health have the plan to compare several Abs for treatment in their ACTIV-2 trial involving outpatients with COVID-19 (NCT04518410).

The protease inhibitors lopinavir, ritonavir and camostat mesylate have been used with the aim to block the activation of S protein by inhibiting the protease TMPRSS2. In order to promote the clinical use of these potential drugs, WHO and the EU have promoted new clinical trials testing the efficacy of drug associations including protease inhibitors, such as the SOLIDARITY Trial (NCT04321616) and the DisCoVeRy Trial (NCT04315948).

A fascinating feature of SARS-CoV-2 is that it could use the S protein not only as a passe-partout to enter cells but also as a signaling ligand to activate intracellular pathways, ERK1/2 among the others, instrumental to replication and evasion of host’s defense. Blocking the S protein with available Abs or interfering with ERK1/2 with inhibitors could be viable ways to suppress the initial steps of cell damage [68]. In line with this, pharmacological inhibition of ERK1/2 or gene knockdown using small interfering RNAs-suppressed coronavirus replication [68,69]. We presented *in vitro* evidence that the S protein alone can elicit functional alterations in pericytes from the human heart, reducing their angiogenic activity and inducing the secretion of pro-inflammatory and -apoptotic factors. These adverse phenomena could be mediated by the S protein interaction with CD147, as neutralization of this receptor by a blocking Ab prevented them [70]. Therefore, the use of receptor entry blocker may exert multiple benefits, reducing the intracellular viral load, viral replication and dampening early inflammatory response. Interventions that block COVID-19 at the early stage could significantly reduce morbidity and mortality, the number of hospitalizations, and the burden on healthcare systems [71].

## Viral replication

Coronaviruses express and replicate their genomic RNA to produce full-length copies that are incorporated into new viral particles (reviewed in [72]). Coronaviruses possess remarkably large RNA genomes that contain cis-acting secondary RNA structures essential for RNA synthesis. At the 5′ end, two large open reading frames (ORFs; ORF1a and ORF1b) occupy a large part of the capped and polyadenylated genome. ORF1a and ORF1b encode nonstructural proteins that are instrumental for viral replication and the transcription complex that includes RNA-processing and -modifying enzymes and an RNA proofreading function necessary for maintaining the integrity of the coronavirus genome.

### Antiviral therapy

Because viral replication is active early during COVID-19, antiviral therapy may exert the greatest benefit before the disease progresses into the hyperinflammatory state of severe disease. Remdesivir, an inhibitor of the viral RNA-dependent RNA polymerase was identified as a promising therapeutic candidate for COVID-19 because of its ability to inhibit SARS-CoV-2 *in vitro* [73]. Moreover, the drug reduced lung virus levels and lung damage in nonhuman primate studies after inoculation with Middle East respiratory syndrome-CoV1 [74]. Currently, remdesivir is the only FDA-approved antiviral drug for the treatment of COVID-19. A double-blind, randomized, placebo-controlled trial of intravenous remdesivir in adults who were hospitalized with COVID-19 and had evidence of lower respiratory tract infection was superior to placebo in shortening the time to recovery [75].

Hydroxychloroquine has antiviral effects *in vitro*, and, in association with azithromycin, seemingly decreased SARS-CoV-2 viral load in a small, nonrandomized study [76]. However, a randomized trial of 504 patients with mild to moderate COVID-19 demonstrated that the use of hydroxychloroquine, alone or with azithromycin, did not improve the clinical status as compared with standard care [77]. Therefore, the current guidelines recommend against the use of this treatment.

### Antibiotics

Emerging data regarding bacterial superinfections in COVID-19 pneumonia suggest an association between the detection of bacterial products in blood and disease severity [78]. Broad-spectrum antibiotics are indicated in these patients with COVID-19 with suspected or confirmed bacterial superinfection [79].

## Inflammation & immune response

The host immune response of SARS-CoV-2 has been a subject of intense investigation (reviewed in [80,81]). The humoral response involves the characteristic IgG and IgM production, starting with Abs against the high immunogenic N protein, while anti-S protein Abs could be detected after 4–8 days from the appearance of initial symptoms [82]. It was reported that a robust Ab response may be associated with disease severity while a weak response is associated with the elimination of the virus [83].

SARS-CoV-2 infection impairs interferon responses and suppresses antigen presentation on both MHC class I and class II, thereby evading the innate immune cells response [84]. The infiltration of monocytes/macrophages, neutrophils and adaptive immune cells leads to increased pro-inflammatory cytokines [85]. A decrease in the innate antiviral response together with hyperinflammation and dysfunction of effector and regulatory T cells characterizes the immune profile of patients with severe COVID-19 [86,87] However, there are also arguments about the concept of COVID-19-related cytokine storm syndrome (COVID-CSS) [88,89]. These criticisms argue that the definition of COVID-CSS is vague, levels of IL-6 (a hallmark of the syndrome) are often low, and some COVID-19 patients have a hypoinflammatory vasculopathy rather than a hyperinflammatory hypercytokinemia syndrome [90]. In order to reconcile these disparities and guide therapeutic decisions, the following criteria have been proposed to identify patients with COVID-CSS: pneumonia requiring mechanical ventilation, fever (maximum temperature >38°C), CRP >100 mg l^-1^ and peak serum ferritin >1000 μg l^-1^ [91]. Patients meeting these criteria had also markedly elevated research serum IL-6 levels, although not always correlated with CRP or ferritin [92].

### Convalescent plasma

Convalescent plasma has been used for the treatment of infectious diseases for more than a century, the rationale being that passive immunization can help to limit the disease severity. To date, it is considered the standard treatment of Argentine hemorrhagic fever, but conclusive data in SARS, Middle East respiratory syndrome, influenza A (H1N1), avian influenza (H5N1), Ebola and eventually COVID-19 are lacking. A trial of 228 patients with severe COVID-19 showed no significant differences in clinical status or overall mortality between patients treated with convalescent plasma and those who received placebo [93]. Similar conclusions were reached in another trial where the convalescent plasma was administered to patients with moderate COVID-19 to halt the progression to severe disease [94]. In January 2021, the RECOVERY trial (NCT04381936) independent Data Monitoring Committee announced that the study investigating the potential benefits of receiving convalescent plasma has stopped assigning people to receive this treatment after an early analysis showed that overall it did not help to reduce deaths. They also decided to continue the recruitment to the tocilizumab treatment arm, and to the other ongoing comparisons – aspirin, colchicine and Regeneron’s Ab cocktail.

### Cytokine inhibitors

The recognition of a state of hypercytokinemia in severe COVID-19 represents a rationale for immunomodulatory and cytokine-inhibitor therapy [95].

The COVACTA RCT (NCT04320615) compared tocilizumab, a humanized monoclonal Ab that blocks IL-6 from binding to receptors, and placebo in COVID-19 reported no difference in the primary outcomes (clinical status and mortality), although a post hoc subanalysis of patients requiring high-flow oxygen by nasal cannula showed an improved clinical status at day 14 [96].

The EMPACTA (Evaluating Minority Patients with Actemra, NCT04372186) placebo-controlled trial demonstrated that tocilizumab could reduce the risk of progression to mechanical ventilation or death among patients receiving low-flow oxygen, but again the active treatment did not improve 28-day survival [97].

The REMAP-CAP (Randomised, Embedded, Multi-factorial, Adaptive Platform Trial for Community-Acquired Pneumonia, NCT02735707) trial found tocilizumab was effective in decreasing inhospital mortality compared with standard care (28% vs 35.8%, adjusted odds ratio for survival 1.64, 95% CI: 1.14–2.35) and progression to intubation, extracorporeal membrane oxygenation or death [98].

Conversely, Veiga *et al.* reported an increased death rate at day 15 in patients treated with tocilizumab compared with placebo (17% vs 3%, odds ratio 6.42, 95% CI: 1.59–43.2), a result that required an early stop of the trial [99]. The reasons for these different outcomes remain unknown.

Altogether, the results of recent trials remain contradictory, possibly because of the wide heterogeneity of inflammatory markers and difficulty to decipher the patient’s phenotype that may benefit from immunomodulation.

### Corticosteroids

Corticosteroids such as dexamethasone (DXM) have been proposed as a potential means to control the complications associated with the cytokine storm.

In the RECOVERY trial, [100] DXM reduced the incidence of death in the group of patients receiving invasive mechanical ventilation (29.3% vs 41.4% in controls) and to a lesser extent in those receiving oxygen without invasive mechanical ventilation (23.3% vs 26.2% in controls). The data also indicated that DXM might increase mortality in hospitalized patients who were not receiving oxygen [100].

A meta-analysis of clinical trials of DXM in patients with severe COVID-19 confirmed that the active treatment with corticosteroids was associated with lower 28-day all-cause mortality compared with usual care or placebo [101]. This meta-analysis comprises pooled data from seven randomized clinical trials of corticosteroids in critically ill patients with COVID-19. The reported mortality was 32.7% in the corticosteroids group and 41.5% in controls. The results were heavily affected by the RECOVERY trial, whose participants represented 59% of total trialed subjects who were included in the meta-analysis.

These landmark trials have informed subsequent practice guidelines for hospitalized patients on supplemental oxygen or mechanical ventilation [102]. Nonetheless, a significant number of patients were unresponsive to DXM and serious adverse events were reported in six of the seven trials of the above meta-analysis, occurring in 18.1% of the patients randomized to corticosteroids and in 23.4% of the patients randomized to usual care or placebo [101].

There are also concerns that, by hindering B cell-mediated Ab production and interfering with the protective function of T cells and macrophage-mediated clearance of apoptotic cells, DXM treatment can result in a higher plasma viral load and an increased risk of secondary infections [103,104]. Therefore, additional therapeutic approaches should be considered for the treatment of patients with severe COVID-19. For instance, it was suggested that future studies may include remdesivir, which was not part of the RECOVERY trial [105].

### Mesenchymal stem cells

The term MSC was officially introduced more than 25 years ago to represent a class of cells from human and mammalian bone marrow and periosteum, that could be isolated and expanded in culture while maintaining the capacity of multilineage differentiation [106,107]. This minimal definition, however, does not reflect the diversity and functional pleiotropism of different MSC populations. MSCs from the stroma of different tissues, like the bone marrow, adipose tissue and the perivascular niche of solid organs, may share a common antigenic phenotype but are also characterized by heterogeneous profiles [108].

#### Previous therapeutic applications of MSCs

More than 1050 clinical trials are registered at FDA.gov that explore MSCs for many clinical applications including neurodegenerative and cardiac disorders, perianal fistulas, Crohn’s disease, graft-versus-host disease, diabetic nephropathy, and organ fibrosis.

About 300 clinical trials using MSCs have been completed as of 2020. Results suggest that the benefit is heterogeneous depending on the modality and purity of the cell preparation and the characteristics of target pathology. The TiGenix/Takeda Phase III clinical trial studying the use of an MSC product (alofisel) for complex perianal fistulas in patients with Crohn’s disease represents the most successful late-stage MSC trial to date (NCT01541579) [109]. In addition to alofisel, there are ten globally approved MSC therapies with various indications including cardiovascular disease (reviewed in [110]). Systematic meta-analyses of trials conducted in patients with myocardial infarction and chronic heart failure showed MSC therapy may improve ventricular function but does not reduce mortality [111,112].

#### Immunological properties & entry receptor expression

The benefit of MSC-based therapies can be reconducted to the paracrine induction of cell repair and the rebalancing of immune cell response in injured or inflamed tissues. Recent review articles have summarized the latest research in the immunological properties of MSCs, their use as immunomodulatory/anti-inflammatory/antimicrobial agents, methods to customize their immunological profile and their use as vehicles for transferring therapeutic agents [113,114].

**Figure 2.**
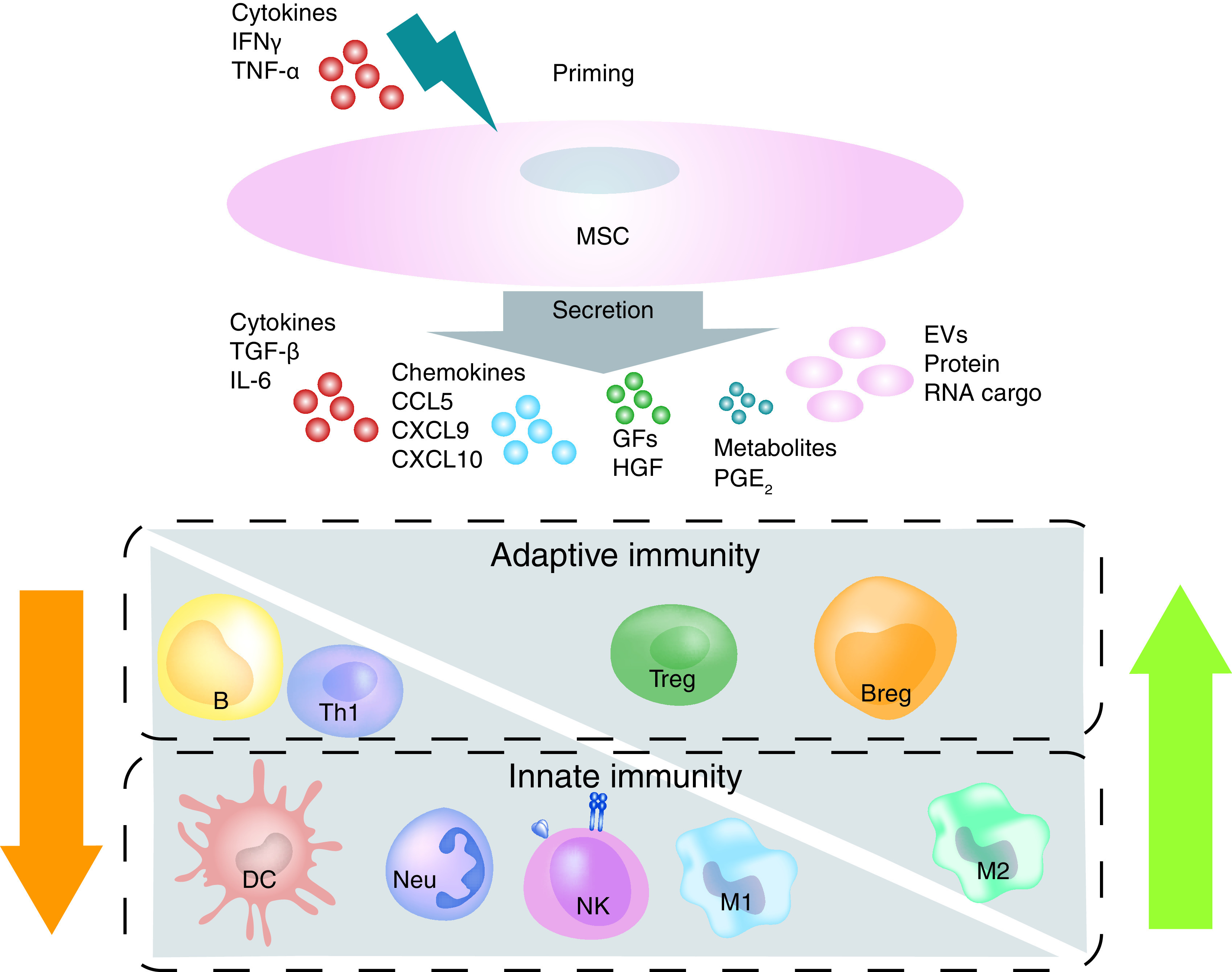
Mesenchymal stem cells modulate both innate & adaptive immune cells. MSCs directly regulate immune cells and this regulation can be influenced by the inflammatory milieu of the host tissue. The immunoregulatory activity of MSCs is exerted mainly via paracrine recruitment and activation of cells from both the innate and adaptive immune systems. Innate: inhibition of NEU and DCs proliferation, NK proliferation and cytotoxic activity, and facilitation of monocytes and macrophages M1–M2 transition. Adaptive: inhibition of Th1 and Th17 and promotion of Treg production, inhibition of B cells via the regulatory Breg. Moreover, MSCs have an additional indirect influence on T and B cells. DC: Dendritic cell; EV: Extracellular vesicle; MSC: Mesenchymal stem cell; NEU: Neutrophil; NK: Natural killer.

Figure 2 illustrates key features of the MSCs’ capacity to modulate innate and adaptive immunity (also reviewed in [115]). Moreover, MSCs can enhance pathogen engulfment by endocytosis and phagocytosis in macrophages [116] as well as improve killing ability in most of these cells. Another important point is the MSC-induced enhancement of efferocytosis of apoptotic or infected cells, which would be extremely important during infection [117].

Noteworthy, another advantage of MSCs is that they do not express the ACE-2 receptor and the priming enzyme that allow SARS-CoV-2 engagement and entry [118]. Cultured MSCs from different tissues were exposed to SARS-CoV-2 wild strain without evidence of cytopathic effects; moreover, under *in vitro* challenges with the virus, the conditioned medium did not contain viral particles [118]. The lack of ACE2 and TMPRSS expression was also interpreted as a key element for the reported clinical success of an MSC therapy trial, described in more detail below, in seven patients with COVID-19 pneumonia [119]. Yet, it should be noted that no direct evidence has been provided so far that MSCs are resistant to infection or maintain an intact functional activity when challenged with the infectious agent in the patient’s body.

#### Preclinical studies using MSC therapy in ARDS

A review from Xiao and colleagues illustrated the results of preclinical research on MSC therapy in models of ARDS and acute lung injury [120]. In the H9N2-infected mouse model, MSC treatment increased the survival rate and decreased lung edema and signs of acute lung injury compared with those of the placebo group [121]. Moreover, cell therapy with MSCs improved gas exchange and reduced the levels of alveolar chemokines and cytokines [121].

Likewise, MSCs were effective in treating an H1N1-infected pig model, reducing viral shedding in nasal swabs and viral replication in the lungs, and lowering the release of proinflammatory cytokines including TNF-α and CXC chemokine ligand 10 [122]. Rogers *et al.* reported the results of studies in animal models and *ex vivo* human lung models showing the MSC’s capacity to inhibit lung damage, reduce inflammation, dampen immune responses and improve alveolar fluid clearance [114].

#### Clinical studies using MSC therapy in ARDS

There have been recent notable studies evaluating the efficacy of MSCs for the treatment of ARDS. The START study was a Phase I pilot trial (NCT01775774) [123], now extended to Phase IIa (NCT02097641). In the Phase I trial, patients were followed daily for adverse events through day 28, death or hospital discharge, whichever occurs first. Vital status was collected at 6 and 12 months after study enrolment. The Phase IIa was a prospective, double-blind, multicenter, randomized trial, comparing a single intravenous dose of cryopreserved bone marrow-derived MSCs of (10 × 10^6^ cells/kg) with placebo in patients with moderate to severe ARDS [124]. The benefit was only marginal, mainly consisting of a reported trend of improved oxygenation in the MSC group.

These findings were in sharp contrast with the results of a clinical study that examined the performance of menstrual blood-derived MSCs for the treatment of 17 critically ill patients with H7N9 influenza induced ARDS [125]. In this case, patients received either three or four infusions of 1 × 10^6^ cells/kg. The MSC group benefitted a remarkably improved survival outcome (54.5% vs 17.6%), with no long-term adverse events being noted. It is not clear whether the difference could be attributed to the methods of preparation and storage (cryopreservation) or the modality of single or repeated injections. It should be noted that studies of this group size are not suited for a definitive conclusion on efficacy.

A systematic literature review and random-effects meta-analysis reported the potential value of MSC therapy in ARDS [126]. MSCs were intravenously or intratracheally administered in 117 participants, who were followed for 14 days to 5 years. All MSCs were allogeneic from bone marrow, umbilical cord, menstrual blood, adipose tissue or unreported sources. No related serious adverse events were reported. Although favorable trends were observed, neither mortality nor functional and biochemical markers were significantly improved by the active treatment.

#### Initial studies of MSC therapy in critical patients with COVID-19

MSCs have been recently trialed for the treatment of severe COVID-19, the main indication being critically patients with the manifestation of ARDS (reviewed in [127,128]).

Small-size trials of MSCs for critically ill COVID-19 patients have been initially conducted in China. A single-center open-label pilot study used MSCs, of an undefined source, to treat seven patients with ARDS in a Beijing Hospital [119]. The disease severity varied among the seven patients studied and only one required mechanical ventilation. All patients receiving MSCs showed clinical improvement after 2 days, and three were discharged from the hospital after 10 days. The authors reported remarkable improvements in inflammatory markers and in the immune cell repertoire, especially Treg and dendritic cells, in treated patients.

A second anecdotical report covered the case of a 65-year-old woman in China treated with three doses of 5 × 10^7^ umbilical cord-derived MSCs. The patient manifested significant clinical improvement, resulting in the cessation of mechanical ventilation, after the second dose, which was matched by a reduction of pneumonia detected in chest CT scans [129].

These results led to an Emergency Use Authorization by the FDA [127]. Conversely, both the International Society for Cellular and Gene Therapies and the International Society for Extracellular Vesicles do not endorse cell products or their subcellular derivatives for any purpose in COVID-19, including but not limited to reducing cytokine storm, exerting regenerative effects or delivering drugs [130].

#### Recent trials of MSC in critical patients with COVID-19

In September 2020, 69 clinical trials utilizing MSCs for the treatment of COVID-19 were registered on the WHO International Clinical Trial Registry Platform [131]. At the time of this review article compilation, a search of the same Registry Platform revealed the number of studies has increased to 103 (Supplementary Table 1). Of these, 49 studies were effectively recruiting, while the remaining 54 were inactive, having been approved but not started yet.

**Figure 3.**
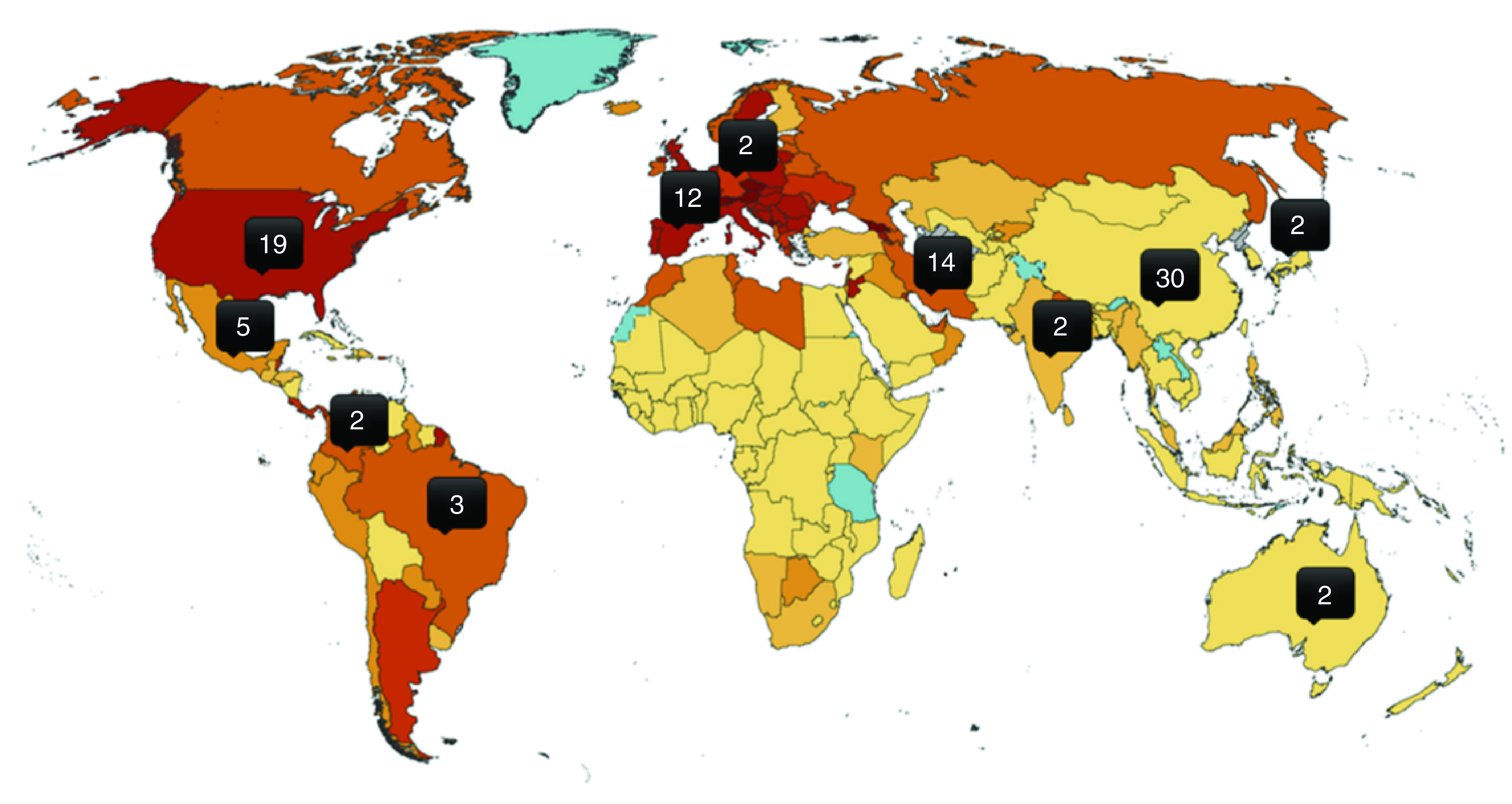
Distribution of clinical trials using mesenchymal stem cells in the world. Only countries with two trials or more are shown.

As shown in Figure 3, most trials are from China (30) followed by the USA (19), Iran (14), Spain (12), Mexico (three) and Brazil (three). Notably, other European countries contributed with only five studies, of which only two are actively recruiting. Considering the declared patient target, the whole 103 studies encompass a population of 4366 patients (mean, 42.3 patient per study), of which 2119 in those that are effectively recruiting (mean, 43.2). In addition, only seven studies have a plan to recruit >100 patients and 30 do not include a control group.

In relation to the tissue of origin for the derivation of MSCs, we could confirm the distribution reported by others in September [131]. Only five trials are investigating MSCs from the bone marrow, the preferred tissue source for other clinical uses. Cord-derived MSCs (including from Wharton’s Jelly) are the most common source with 37 trials, followed by 15 from adipose tissue, five from dental pulp and four from the placenta, while 18 were using MSCs of undefined origin. A few trials employ either MSC-conditioned media or vesicles/exosomes, which are acknowledged to exert similar immunomodulatory and reparative activities of the cells from which they are derived [132–134].

#### Personal considerations on current trial methodology

In a critical situation like the current pandemic, all possible solutions with the potential to alleviate the consequences of COVID-19 merit consideration. It is currently premature to draw conclusions about the efficacy and safety of MSC trials as most of them are still ongoing. The current challenge in COVID-19 therapeutics, including MSC therapy, is the quality of trial studies, the imbalance of their subjects, and the quality and robustness of preliminary reports [135].

A view of Supplementary Table 1 summarizing MSC trials clearly indicates that these studies differ greatly from each other about preparation, dosage (from a few million to a hundred million), administration schedule (single or repeated dosage) and the best combination with other anti-inflammatory agents. In addition, there is a large variation on primary end points. Ten out of the 49 trials currently recruiting participants have safety as a primary end point, alone or in combination with efficacy. Thirteen (of which 11 are controlled trials) focus on mortality. The remaining trials assess softer end points such as clinically, laboratory or imaging data (mainly blood oxygen saturation and CT scan of the lungs).

The current scenario does not differ from the typical stem cell therapy landscape: a plethora of small size trials that are unlikely to provide definitive conclusions, due to the diversity of cell source, dosages and protocols of administration. Unfortunately, these drawbacks can also preclude the applicability of meta-analyses (often incorrectly employed with the hope to amend the initial errors), due to concerns regarding statistical power and confusion from two major sources of variation: pitfalls in trial design and inconsistencies in reporting and interpreting trial results.

In addition, in the authors’ opinion, several caveats reduce the enthusiasm for the cell therapy approach. First, unless MSC-based therapy demonstrates to be effective in patients unresponsive to DXM, corticosteroids remain formidable competitors due to the much lower cost and more flexible dosage. Second, no biomarker exists to predict the safety and efficacy of MSCs in COVID-19. The cytokine storm profile differs among patients affected by severe COVID-19, this being, as mentioned above, a burden also for the proper use of cytokine-targeting inhibitors [136]. Third, after intravenous injection, MSCs are captured in the capillary bed of the lungs. Here, they are supposed to reduce inflammation and restore endothelial integrity [137], yet this mechanism may be dysfunctional in COVID-19 patients with ARDS due to the severe microvascular damage favoring clotting of infused cells. In fact, one of the few identified complications is the risk of MSC therapy-induced thrombosis, reported in several patients before the insurgence of COVID-19 [138–140]. A recent clinical trial showed that intravenous infusion of allogeneic adipose tissue-derived MSCs exerted mixed pro- and anti-inflammatory as well as procoagulant effects during human endotoxemia [141]. The procoagulant activity of MSCs was associated with a mechanism involving phosphatidylserine and tissue factor, which requires further analysis to avoid adverse effects of MSC therapy in patients with a risk of thrombosis [142]. Finally, while ARDS remains the main clinical indication for the cellular approach, there is no experimental evidence that supports the utilization of MSCs for the treatment of cardiac complications of COVID-19, an extended application that might be instigated by previous experience of cell therapy in patients with myocardial infarction or heart failure [143].

#### Commercial MSC trials for COVID-19

Cell therapies are advanced therapy medicinal products. Their development according to the highest quality standards and needs for off-the-shelf deployment is preferentially achieved through a commercial route. Therefore, an overview of MSC trials conducted by companies may help to gauge the validity of this approach.

Several companies are repurposing their MSC products for therapeutic use in COVID-19. For example, Mesoblast Limited (Nasdaq: MESO; ASX: MSB), a global leader in allogeneic cellular medicines for inflammatory diseases, has recently proposed the use of ryoncil^®^ (remestemcel-L) to treat patients with moderate to severe ARDS. The compound is currently under priority review by the FDA for steroid-refractory acute graft-versus-host disease. Pilot data indicate that survival rate was 83% in ventilator-dependent COVID-19 patients when treated with two intravenous infusions of remestemcel-L, whereas the survival rate was only 12% in those receiving standard of care during the same period. Remestemcel-L is believed to counteract the pathological process by downregulating the production of pro-inflammatory cytokines and increasing the production of anti-inflammatory cytokines.

To confirm these pilot data, Mesoblast has launched a Phase III randomized controlled trial (NCT04371393) of up to 300 ventilator-dependent adults with moderate or severe COVID-19 ARDS. The dosing regimen in Phase III is the same as in the pilot trial and the end point is the reduction in mortality. The Data Safety Monitoring Board will perform an interim analysis of the trial’s primary end point of all-cause mortality within 30 days of randomization. Further interim analysis is planned after 60% of the trial has been enrolled.

It is not surprising that leading companies are pursuing an accelerated approval pathway for their advanced cell products. They have already robust manufacturing and quality control data, preclinical evidence of safety and efficacy, and an adequate budget for preclinical and clinical experimentation. Their return is potentially massive and commensurate to the pandemic impact of COVID-19. It is estimated that 25% of hospitalized patients require intensive treatment and 1% develop severe COVID-19. Projected to the total number of cases and considering a US $4000 cost for a single MSC treatment, the total cost to treat previous cases would have been equivalent to $2320 billion.

## Conclusion

As massive vaccination programs against SARS-CoV-2 are now ongoing, the open question is how to manage newly infected patients suffering from COVID-19 and whether immunization will be sufficient to eradicate the problem. It is, therefore, crucial to implement different treatments and refine current guidelines according to the severity and stage of the disease. While the pilot studies showed a promising stance for some of these products, large and well-designed trials are warranted. In addition, preclinical research in cellular and animal models should define a stronger rationale in support of clinical experimentation. Additional investigation is also needed on biomarkers guiding the choice of anti-inflammatory and immunomodulatory drugs.

## Future perspective

There are important lessons from the COVID-19 pandemic. The first lesson is that the coronavirus does not respect national boundaries. Therefore, everyone in the scientific community bears great responsibility for collaborating and sharing results, with great attention to the ethics and robustness of the provided evidence.

On the one hand, we need that data are collected and analyzed rapidly. On the other hand, it is crucial that researchers take primary responsibility for the production and use of knowledge making sure about the quality of basic, translational, and clinical studies and related reports. This review contains many references that are preprint articles, which have not received regular scrutiny through referees’ evaluation. Readers need to be aware of the limitations of this new method to communicate the results. These challenges are highlighted by a recent article illustrating how the health research system may have to deal with the inevitable imperfections of rapid scientific reporting in the current and future crises [144].

The second lesson has medical, commercial and governmental implications. It regards the pressing need for global-readiness programs aiming to develop treatments and vaccines that can mitigate future viral pandemics [145]. Developing novel antiviral agents including blocking Abs, antiviral drugs and vaccines is financially costly. Creating vaccines, especially under the pressure of an acute health emergency, has not proved very rewarding in the past, meaning pharmaceutical companies may be reluctant in investing their budgets in such long-term programs. Therefore, governments should continue to invest in pandemic strategies the same way they do now in defense. In the USA alone, deaths due to SARS-CoV-2 have reached today (3 February 2021) 447,000, a figure that surpasses the 405,399 losses that occurred during World War II. Academic institutions should participate and/or lead these global-preparedness endeavors. Repurposing clinically available drugs, as exemplified by the use of corticosteroids in COVID-19, could be cost/benefit advantageous.

The third lesson is that COVID-19 has prompted a remarkable change in medical practice, providing the battlefield for a new army of doctors and nurses to nurture experience in capturing critical needs in real-time selecting options from a growing armamentarium of approved medicines and support devices. In the future, machine learning technologies could be harnessed to help to integrate scientific and medical knowledge into rapid diagnostic flowcharts and personalized treatments.

**Executive summary**

**Epidemiology & emergence of new variants**

- As of 3 February 2021, over 103 million cases of COVID-19 have been reported, including 2.24 million deaths. Severe lung disease characterized by acute respiratory distress syndrome (ARDS) represents the most severe complication. Multiple variants of the virus that causes COVID-19 are emerging globally.

**Clinical presentation**

- COVID-19 can present a variety of manifestations ranging from asymptomatic infection to critical disease. No precise guidelines for classification have been established, although the current definition refers to mild, moderate and critical disease with or without ARDS. Treatment protocols follow this classification as well as what is known about target pathogenic mechanisms during disease progression. The S protein is the leading mediator of viral entry following processing by host cell protease.

**Anchor receptors**

- The first target for therapy of COVID-19 is shielding epithelial and endothelial cells from the virus contact.

**Conventional & unconventional entry receptors**

- The second target is to interfere with entry receptor engagement.

**Viral replication**

- The third target is to use antiviral agents to inhibit viral replication together with antibiotics if superinfection occurs.

**Inflammation & immune response**

- Anti-inflammatory therapy and immunomodulatory drugs are indicated in severe disease to combat the state of hyperinflammation and cytokine storm together with evasion of the immune response. Different approaches include the use of convalescent plasma, cytokine inhibitors, steroids and immunomodulatory stem cells. The latter have been trialed in small studies and now examined in patients with ARDS. Pharmaceutical companies have repurposed their approved cell products to this clinical application.

**Conclusion**

- The massive vaccination campaign will not resolve the problem entirely and the above approaches need to be further refined and incorporated in an approved protocol.

**Future perspective**

- We have learned lessons that will inform future decisions at the level of research, communication and readiness plans to face new emergencies.
