# Supplementary material for: Treatment of COVID-19 by stage: any space left for mesenchymal stem cell therapy?
Source: Regen Med. 2021 May 14:10.2217/rme-2020-0189. doi: 10.2217/rme-2020-0189 (PMC8127835; doi:10.2217/rme-2020-0189)
Supplement: Supplementary file 1 [file Supplementary_Table_1.docx]

| Trial ID | Scientific title | Country | Source / Dose | Participants | |
| --- | --- | --- | --- | --- | --- |
| EUCTR2020-001505-22-ES | Double-blind, randomized, parallel, placebo-controlled pilot clinical trial, nested in a prospective cohort observational study, for the evaluation of the efficacy and safety of two doses of WJ-MSC in patients with acute respiratory distress syndrome secondary to infection by COVID-19 - COVIDMES | Spain | Umbilical cord  **Dose:** No information available | 15 Control 15 Exp | |
| ChiCTR2000030173 | Key techniques of umbilical cord mesenchymal stem cells for the treatment of novel coronavirus pneumonia (COVID-19) and clinical application demonstration | China | Umbilical cord  **Dose:** no information available | 30 Control 30 Exp | |
| ChiCTR2000030138 | Clinical Trial for Human Mesenchymal Stem Cells in the Treatment of Severe Novel Coronavirus Pneumonia (COVID-19) | China | Umbilical cord  **Dose:** IV injection, no information available | 30 Control 30 Exp | |
| ChiCTR2000030088 | Umbilical cord Wharton's Jelly derived mesenchymal stem cells in the treatment of severe novel coronavirus pneumonia (COVID-19) | China | Wharton's Jelly  **Dose:** IV injection of mesenchymal stem cells (1*10E6/kg), cell suspension volume | 20 Control 20 Exp | |
| ChiCTR2000030261 | A study for the key technology of mesenchymal stem cells exosomes atomization in the treatment of novel coronavirus pneumonia (COVID-19) | China | MSC Exosomes (origin not specified)  **Dose:** Aerosol inhalation of exosomes | 13 Control 13 Exp | |
| EUCTR2019-002688-89-ES | Clinical Study to Assess the Safety and Preliminary Efficacy of HCR040 in Acute Respiratory Distress Syndrome | Spain | Adipose tissue, HCR040® cells  **Dose:** IV, No information available | 14 Control 14 Exp | |
| EUCTR2020-001682-36-ES | Treatment of COVID-19 with allogeneic mesenchymal cells (MSVÂ®) | Spain | Allogeneic mesenchymal cells, MSV®  **Dose:** No information available | 12 Control 12 Exp | |
| EUCTR2020-001266-11-ES | Clinical trial of administration of MSC to patients with respiratory distress type COVID-19 | Spain | Adipose tissue  **Dose:** No information available | 50 Control 50 Exp | |
| NCT04377334 | Prospective Phase II Study: MSCs in Inflammation-Resolution Programs of SARS-CoV-2 Induced ARDS | Germany | Bone marrow  **Dose:** No information available | 20 Control 20 Exp | |
| EUCTR2020-001364-29-ES | Study with stem cells from allogenic adipose tissue, in patients with coronavirus severe pneumonia | Spain | Adipose tissue  **Dose:** Allogenic cells, no information available | 13 Control 13 Exp | |
| ChiCTR2000029817 | Clinical Study of Cord Blood NK Cells Combined with Cord Blood Mesenchymal Stem Cells in the Treatment of Acute Novel Coronavirus Pneumonia (COVID-19) | China | Cord blood  **Dose:** High dose group: High-dose NK cells (>5*10E9)and mesenchymal stem cells(>5*10E9), Intravenous infusion once every two days for a total of five times; Conventional dose group: Conventional dose NK cells (>3*10E9) and mesenchymal stem cells(>3*10E9),Intravenous infusion once every two days for a total of three times; Preventive dose group: Preventive dose NK cells (>3*10E9)and mesenchymal stem cells(>3*10E9),Intravenous infusion once every week for a total of one time; | 60 Exp | |
| ChiCTR2000029816 | Clinical Study of Cord Blood Mesenchymal Stem Cells in the Treatment of Acute Novel Coronavirus Pneumonia (COVID-19) | China | Cord blood  **Dose:** No information available | 30 Control 30 Exp | |
| NCT04349631 | A Clinical Trial to Determine the Safety and Efficacy of Hope Biosciences Autologous Mesenchymal Stem Cell Therapy (HB-adMSCs) to Provide Protection Against COVID-19 | USA | Adipose tissue, HB-adMSCs  **Dose:** Five IV infusion of autologous adipose-derived mesenchymal stem cells. | 56 Exp | |
| NCT04302519 | Clinical Study of Novel Coronavirus Induced Severe Pneumonia Treated by Dental Pulp Mesenchymal Stem Cells | China | Dental Pulp  **Dose:** No information available | 24 Exp | |
| ChiCTR2000030224 | Clinical study of mesenchymal stem cells in treating severe novel coronavirus pneumonia (COVID-19) | China | Cord blood  **Dose:** No information available | 30 Control 30 Exp | |
| ChiCTR2000031319 | Safety and Efficacy Study of Allogeneic Human Dental Pulp Mesenchymal Stem Cells to Treat Severe Pneumonia of COVID-19:a Single-center, Prospective, Randomised Clinical Trial | China | Dental pulp  **Dose**: IV, no information available | 10 Control 10 Exp | |
| IRCT20140528017891N8 | Evaluation of the efficacy and safety of cord-derived mesenchymal stem cell transplantation in the treatment of COVID-19 | Iran | Umbilical cord  **Dose:** IV 0.5-1*10E6/ kg body weight on the first, third and sixth days. | 5 Control 5 Exp | |
| IRCT20200325046860N2 | Mesenchymal stem cell utilization in reducing complications and enhancing pneumonia healing in patients infected with 2019-nCoV (phase I clinical trial) | Iran | Not specified  **Dose:** IV 7*10E6 cells at day 0, 3 6 | 5 Exp | |
| NCT04273646 | Clinical Study of Human Umbilical Cord Mesenchymal Stem Cells in the Treatment of Severe COVID-19 | China | Umbilical cord  **Dose**: IV transfusion one round (4 times) of 5.0*10E6 cells/kg of UC-MSCs | 24 Control 24 Exp | |
| NCT04346368 | Safety and Efficacy of Intravenous Infusion of Bone Marrow-Derived Mesenchymal Stem Cells in Severe Patients With Coronavirus Disease 2019 (COVID-19): A Phase 1/2 Randomized Controlled Trial | China | Bone marrow  **Dose:** IV 1*10E6 /kg body weight at Day 1 | 10 Control 10 Exp | |
| NCT04348461 | Two-treatment,Randomized, Controlled, Multicenter Clinical Trial to Assess the Safety and Efficacy of Intravenous Administration of Expanded Allogeneic Adipose Tissue Adult Mesenchymal Stromal Cells in Critically Ill Patients COVID-19 | Spain | Adipose tissue  **Dose:** Two serial doses of 1.5 million cells/ kg body weight | 100 Exp | |
| NCT04352803 | IV Infusion of Autologous Adipose Derived Mesenchymal Cells for Abatement of Respiratory Compromise in SARS-CoV-2 Pandemic (COVID-19) | Spain USA | Adipose tissue  **Dose:** IV infusion of autologous cells, no information available | 10 Control 10 Exp | |
| NCT04366830 | Intermediate-size Expanded Access of Remestemcel-L, Ex-vivo Cultured Adult Human Mesenchymal Stromal Cells for Acute Respiratory Distress Syndrome Due to COVID-19 Infection | USA | Not specified, Remestemcel-L  **Dose:** Remestemcel-L, two IV infusions of 2 * 10E6 / kg body weight | 50 Exp | |
| NCT04371601 | Safety and Effectiveness of Mesenchymal Stem Cells in the Treatment of Pneumonia of Coronavirus Disease 2019 | China | Umbilical cord  **Dose:** 10E6 / Kg body weight / time, once every 4 days for a total of 4 times | 30 Control 30 Exp | |
| NCT04341610 | Allogeneic Adipose Tissue Derived Mesenchymal Stromal Cell Therapy for Treating Patients With Severe Respiratory COVID-19. A Danish, Double-blind, Randomized Placebo-controlled Study | Denmark | Adipose tissue  **Dose:** 100*10E6 cells | 20 Control 20 Exp | |
| IRCT20200217046526N1 | Mesenchymal Stem Cell Therapy for Acute Respiratory Distress Syndrome in Coronavirus Infection : A Phase 1 and 2 clinical trial | Iran | Not specified  **Dose:** IV, three doses of 200*10E6 cells at day 0, day 2, day 4. | 6 Exp | |
| NCT04315987 | Exploratory Clinical Study to Assess the Efficacy of NestaCellÂ® Mesenchymal Stem Cell to Treat Patients With Severe COVID-19 Pneumonia | Brazil | NestCell® MSCs  **Dose**: IV 2*10E7 cells on days 1, 3, 5 and 7 | 45 Control 45 Exp | |
| NCT04429763 | Safety and Efficacy of Mesenchymal Stem Cells in the Management of Severe COVID-19 | Colombia | Umbilical cord  **Dose:** 1*10E6 cells/Kg body weight | 15 Control 15 Exp | |
| IRCT20200426047206N2 | Clinical trial of efficacy and safety of mesenchymal stem cell transplantation in patients with COVID-19 pneumonia | Iran | Umbilical cord  **Dose:** 1*10E6 cells/Kg body weight | 15 Control 15 Exp | |
| NCT04390152 | Mesenchymal Stem Cell Plus Standard Therapy for the Treatment of Patients With Acute Respiratory Distress Syndrome Diagnosis Due to COVID 19: A Randomized Controlled Trial | Colombia | Wharton's jelly  **Dose:** 50*10E6 cells, two doses | 20 Control 20 Exp |  |
| NCT04467047 | Safety and Feasibility of Allogenic Mesenchymal Stromal Cells in the Treatment of COVID-19 | Iran | Umbilical cord  **Dose:** IV 1*10E6 cells | 15 Control 15 Exp |  |
| NCT04456361 | A Study of Mesenchymal Stem Cells as a Treatment in Patients With Acute Respiratory Distress Syndrome Caused by COVID-19 | Mexico | Wharton Jelly  **Dose:** IV single-dose 1 * 10E8 cells | 9 Exp |  |
| NCT04288102 | A Phase II, Multicenter, Randomized, Double-blind, Placebo-controlled Trial to Evaluate the Efficacy and Safety of Human Umbilical Cord-derived Mesenchymal Stem Cells in the Treatment of Severe COVID-19 Patients | China | Umbilical cord  **Dose:** IV transfusion 3 times of MSCs (4.0*10E7 cells per time) | 30 Control 60 Exp |  |
| NCT03042143 | Repair of Acute Respiratory Distress Syndrome by Stromal Cell Administration (REALIST): An Open Label Dose Escalation Phase 1 Trial Followed by a Randomized, Double-blind, Placebo-controlled Phase 2 Trial (COVID-19) | UK | Umbilical cord, Realist Orbcel-C CD362 enriched MSCs  **Dose:** dose escalation pilot study in which cohorts of subjects with moderate to severe ARDS will receive increasing doses of a single infusion of Realist Orbcel-C in a 3+3 design. Initially 3 cohorts with 3 subjects/cohort. i | 9 Exp followed by 75 Exp |  |
| NCT04348435 | A Randomized, Double-Blind, Placebo-Controlled Clinical Trial to Determine the Safety and Efficacy of Hope Biosciences Allogeneic Mesenchymal Stem Cell Therapy (HB-adMSCs) to Provide Protection Against COVID-19 | USA | Adipose tissue, HB-adMSCs  **Dose:** IV, Allogeneic cells 200*10E6  5 Infusions at weeks 0, 2, 6, 10, and 14. | 50 Controls 50 Exp |  |
| NCT04522986 | An Exploratory Study of ADR-001 in Patients With Severe Pneumonia Caused by SARS-CoV-2 Infection | Japan | Adipose tissue  **Dose:** IV 1*10E8 cells are administered once a week, total four times | 6 Exp |  |
| NCT04276987 | A Pilot Clinical Study on Aerosol Inhalation of the Exosomes Derived From Allogenic Adipose Mesenchymal Stem Cells in the Treatment of Severe Patients With Novel Coronavirus Pneumonia | China | Adipose Tissue—MSC Exosomes  **Dose**: 5 times aerosol inhalation of MSCs-derived exosomes (2.0*10E8 nano vesicles/3 ml at Day 1, Day 2, Day 3, Day 4, Day 5). | 24 Exp |  |
| NCT04490486 | Phase I, Randomized, Double Blinded, Placebo Control Study to Evaluate the Safety and Potential Efficacy of Intravenous Infusion of Umbilical Cord Tissue (UC) Derived Mesenchymal Stem Cells (MSCs) Versus Placebo to Treat Acute Pulmonary Inflammation Due to COVID-19 With Moderate to Severe Symptoms | USA | Umbilical cord  **Dose:** IV 100 * 10E6 cells at day 0 and 3 | 10 Control 10 Exp |  |
| NCT04527224 | A Phase I/?a Trial to Explore the Safety and Efficacy of Allogenic Adipose Tissue-derived Mesenchymal Stem Cell (AstroStem-V) Therapy in Patients With COVID-19 Pneumonia | China | Adipose tissue AstroStem-V  **Dose:** no information available | 10 Exp |  |
| ChiCTR2000029569 | Safety and efficacy of umbilical cord blood mononuclear cells conditioned medium in the treatment of severe and critically novel coronavirus pneumonia (COVID-19): a randomized controlled trial | South Corea | Conditioned Media from Umbilical Cord MSCs  **Dose:** no information available | 15 Control 15 Exp |  |
| NCT04452097 | A Phase 1 Study of the Safety and Tolerability of BX-U001 for the Treatment of Severe COVID-19 Pneumonia With Moderate to Severe Acute Respiratory Distress Syndrome (ARDS). | USA | Umbilical cord  **Dose:** single IV BX-U001 at 0.5*10E6, 1.0*10E6, or 1.5*10E6 cells/kg of body weight | 9 Exp |  |
| JPRN-JapicCTI-205465 | Umbilical cord-derived mesenchymal stromal cells therapy for SARS-CoV-2 infection (COVID-19) related Acute Respiratory Distress Syndrome | Japan | Umbilical cord  **Dose:** IV 2*10E6 cells / kg body weight or 1*10E6 cells/kg body weight once a day two days apart in between. One cycle is defined as 2 administraitons and patient will be treated with total 2 cycles (4 administrations). | 12 Exp |  |
| NCT04573270 | A Pilot Phase Study Evaluating the Effects of a Single Mesenchymal Stem Cell Injection in Patients With Suspected or Confirmed COVID-19 Infection and Healthcare Providers Exposed to Coronavirus Patients | USA | Umbilical cord  **Dose:** No information available | 40 Exp |  |
| NCT04445220 | A Multi-center, Randomized, Case Controlled, Double-blind, Ascending-dose Study of Extracorporeal Mesenchymal Stromal Cell Therapy (SBI-101 Therapy) in COVID-19 Subjects With Acute Kidney Injury Receiving Renal Replacement Therapy | USA | SBI-101 is a biologic/device combination product that combines two components: allogeneic human MSCs and an FDA-approved plasmapheresis device  **Dose:** SBI-101 device containing 250 * 10E6 MSCs SBI-101 device containing 750 * 10E6 MSCs | 11 Control 11 Exp |  |
| NCT04456439 | Intermediate-size Expanded Access of Remestemcel-L, Human Mesenchymal Stromal Cells, for Multisystem Inflammatory Syndrome in Children (MIS-C) Associated With Coronavirus Disease (COVID-19) | USA | Not specified  **Dose:** 2 infusions of 2 *10E6 remestemcel-L within a 5-day period. | 50 Exp |  |
| NCT04535856 | Therapeutic Study to Evaluate the Safety and Efficacy of DW-MSC in COVID-19 Patients: Randomized, Double-blind, and Placebo-controlled | Indonesia | Not specified, DW-MSC  **Dose:** Low-dose group (5* 10E7cells), 2 vials  High-dose group (5* 10E8cells),4 vials | 3 Control 6 Exp |  |
| CTRI/2020/08/027043 | A Phase 1 clinical trial of intravenous administration of mesenchymal stem cells derived from umbilical cord and placenta in patients with novel COVID-19 virus pneumonia. | India | Umbilical cord and placenta  **Dose:** 100 * 10E6 cells in 10 patients and 100 * 10E6 cells in 10 patients in two doses on day 1 and day 4 | 20 Exp |  |
| CTRI/2020/10/028250 | A Randomized, Controlled, Open Label, Multicentre, Two Arm, Two Dosage, Phase II Study Assessing the Efficacy and Safety of Intravenous Administration of Adult Human Bone Marrow Derived, Cultured, Pooled, Allogeneic Mesenchymal Stromal Cells in Patients with Acute Respiratory Distress Syndrome Caused by Pneumonia due to COVID-19 | India | Bone Marrow  **Dose:** Ex - vivo cultured allogeneic Mesenchymal stromal cells at a dose of 200 *10E6 cells at Day 0 and Day 3 | 20 Control 20 Exp |  |
| RBR-3fz9yr | Use of mesenchymal cells for the treatment of patients with severe acute respiratory syndrome caused by SARS-CoV-2 - : Coronavirus infections | Brazil | Umbilical cord  **Dose:** Three doses of 500.000 cells/kg body weight | 5 Control 10 Exp |  |
| NCT04345601 | Single Donor Banked Bone Marrow Mesenchymal Stromal Cells for the Treatment of COVID19-Induced ARDS: A Randomized, Controlled Study | USA | Bone Marrow  **Dose:** IV injection,1 x 10^8 cells | 30 Exp |  |
| NCT04355728 | Umbilical Cord-derived Mesenchymal Stem Cells for COVID-19 Patients With Acute Respiratory Distress Syndrome (ARDS) | USA | Umbilical cord  **Dose:** IV, 100*10E6 cells at 1 and 3 day | 12 Control 12 Exp |  |
| NCT04428801 | Clinical Study for the Prophylactic Efficacy of Autologous Adipose Tissue-Derived Mesenchymal Stem Cells (AdMSCs) Against Coronavirus 2019 (COVID-19) | USA | Adipose tissue  **Dose:** IV three doses of 200 * 10E6 cells every 3 days | 100 Control 100 Exp |  |
| NCT04625738 | Efficacy of Infusions of Mesenchymal Stem Cells From Wharton Jelly in the Moderate to Severe SARS-Cov-2 Related Acute Respiratory Distress Syndrome (COVID-19): A Phase IIa Double-blind Randomized Controlled Trial | USA | Wharton Jelly  Dose: Day 0 (or 1): 1*10^6 MSC/kg (maximum 80*10^6 MSC)  Day 3 (or 4): 0.5 *10^6 MSC/kg (maximum 40* 10^6 MSC)  Day 5 (or 6): 0.5*10^6 MSC/kg (maximum 40* 10^6 MSC) An interval of 2 days will be respected between 2 infusions. | 15 Control 15 Exp |  |
| ChiCTR2000030484 | HUMSCs and Exosomes Treating Patients with Lung Injury following Novel Coronavirus Pneumonia (COVID-19) | China | Umbilical Cord + Exosomes  **Dose**: Group 1, IV infusion 1: 5 *10E7 cells / time, once / week, twice / course  group 2: IV infusion, 5 * 107 cells / time, 1 time / week, 2 times / course, a total of 2 courses; Exosomes: IV administration, 180mg / time, 1 time / day, 7 days / course, 2 courses in total | 30 Control 30 Exp |  |
| Recruiting trials | **Scientific title** | **Country** | **Source / Dose/ primary endpoint** | **Participants** |  |
| ChiCTR2000030116 | Safety and effectiveness of human umbilical cord mesenchymal stem cells in the treatment of acute respiratory distress syndrome of severe novel coronavirus pneumonia (COVID-19) | China | Umbilical Cord  **Dose:** no information available  **Primary Outcome(s):** Time to leave ventilator on day 28 | 16 Exp |  |
| NCT04269525 | Umbilical Cord(UC)-Derived Mesenchymal Stem Cells(MSCs) Treatment for the 2019-novel Coronavirus (nCOV) Pneumonia | China | Umbilical Cord  **Dose:** Different stem cell doses  **Primary Outcome(s):** Oxygenation index [Time Frame: on the day 14 after enrollment] | 10 Exp |  |
| ChiCTR2000030020 | The clinical application and basic research related to mesenchymal stem cells to treat novel coronavirus pneumonia (COVID-19) | China | Not Specified  **Primary Outcome(s):** Coronavirus nucleic acid markers negative rate; Symptoms improved after 4 treatments; Inflammation (CT of the chest) | 20 Exp |  |
| ChiCTR2000029990 | Clinical trials of mesenchymal stem cells for the treatment of pneumonitis caused by novel coronavirus (COVID-19) | China | Not specified  **Dose:** no information available  **Primary Outcome(s):** Improved respiratory system function (blood oxygen saturation) recovery time | 60 Control 60 Exp |  |
| ChiCTR2000029580 | Severe novel coronavirus pneumonia (COVID-19) patients treated with ruxolitinib in combination with mesenchymal stem cells: a prospective, single blind, randomized controlled clinical trial | China | MSCs (origin not specified) + Ruxolitinib  **Dose**: No information available  **Primary Outcome(s):** Safety | 35 Control 35 Exp |  |
| ChiCTR2000030866 | Open-label, observational study of human umbilical cord derived mesenchymal stem cells in the treatment of severe and critical COVID-1 | China | Umbilical cord  **Dose:** IV infusion of 1×10^6 UCMSCs/kg/time on day 0, 3, 6  **Primary Outcome(s):** Oxygenation index (arterial oxygen partial pressure (PaO2) / oxygen concentration (FiO2)); Conversion rate from serious to critical patients; Conversion rate and time from critical to serious patients; Mortality in serious and critical patients | 30 Exp |  |
| ChiCTR2000030835 | Clinical study on the efficacy of Mesenchymal stem cells (MSC) in the treatment of severe novel coronavirus pneumonia (COVID-19) | China | Umbilical cord  **Dose**: MSc (2 x10^6 / kg / time) or MSc (1x10^6 / kg / time)  **Primary Outcome(s):** C-reactive protein; Detection of lymphocyte subsets; Procalcitonin; Routine blood test; Chest CT;cytokine; Blood biochemistry; | 20 Exp |  |
| NCT04313322 | Treatment of COVID-19 Patients Using Wharton's Jelly-Mesenchymal Stem Cells | Jordan | Wharton's Jelly—MSCs  **Dose:** No information available  **Primary Outcome(s):** Clinical outcome [Time Frame: 3 weeks]  CT Scan [Time Frame: 3 weeks]  RT-PCR results [Time Frame: 3 weeks] | 5 Exp |  |
| ChiCTR2000031494 | Clinical study for stem cells in the treatment of severe novel coronavirus pneumonia (COVID-19) | China | Umbilical cord  **Dose:** IV infusion, no information available  **Primary Outcome(s):** Chest Imaging; lung function;ADL | 18 Control 18 Exp |  |
| ChiCTR2000031430 | Evaluation of the safety and efficacy for human umbilical cord mesenchymal stem cells in COVID-19 induced pulmonary fibrosis | China | Umbilical cord  **Dose:** No information available  **Primary Outcome(s):** Laboratory tests | 100 Control 100 Exp |  |
| ChiCTR2000029606 | Clinical Study for Human Menstrual Blood-derived Stem Cells in the Treatment of Acute Novel Coronavirus Pneumonia (COVID-19) | China | Human Menstrual Blood-derived Stem Cells preparations  **Dose:** IV infusion of cells, no information available  Artificial liver therapy with or without Human Menstrual Blood-derived Stem Cells preparations  **Dose:** IV infusion of cells, no information available  **Primary Outcome(s):** Mortality in patients | 15 Control 18 Exp  10 Control 10 Exp/10 Exp |  |
| NCT04336254 | Safety and Efficacy Study of Allogeneic Human Dental Pulp Mesenchymal Stem Cells to Treat Severe Pneumonia of COVID-19:a Single-center, Prospective, Randomised Clinical Trial | China | Dental pulp  **Dose:** IV injection of 3.0x10e7 cells solution (30ml) on day 1, day 4 and day 7  **Primary Outcome(s):** TTCI [Time Frame: 1-28 days] | 10 Control 10 Exp |  |
| NCT04339660 | Clinical Research of Human Mesenchymal Stem Cells in the Treatment of COVID-19 Pneumonia | China | Umbilical cord  **Dose:** IV 1*10E6 UC-MSCs/kg body weight in a single dose eventually repeated depending on the condition of the need to be given again at an interval of 1 week.  **Primary Outcome(s):** Blood oxygen saturation, immune function | 15 Control 15 Exp |  |
| ChiCTR2000030300 | Umbilical cord mesenchymal stem cells for the treatment of patients at high risk of novel coronavirus pneumonia (COVID-19): a single-center, prospective, open clinical study | China | Umbilical cord  **Dose:** No information available  **Primary Outcome(s):** Time to disease recovery; Exacerbation (transfer to RICU) time | 9 Exp |  |
| IRCT20140911019125N6 | Study the effect of intravenous injection of dental pulp mesenchymal stem cells in treatment of patients with COVID-19 pneumonia | Iran | Dental pulp  **Dose:** IV 1*10E6 cells  **Primary Outcome(s):** Expression of nucleic acid of virus, Lymphocytes count, Patients clinical sign, Pulmonary Conditions at TC scan | 10 Exp |  |
| NCT04252118 | Safety and Efficiency of Mesenchymal Stem Cell in Treating Pneumonia Patients Infected With COVID-19 | China | Not specified  **Dose:** IV, 3 times of MSCs (3.0*10E7 MSCs intravenously at Day 0, Day 3, Day 6).  **Primary Outcome(s):** Side effects in the MSCs treatment group, Size of lesion area by chest radiograph or CT | 10 Control 10 Exp |  |
| NCT04366063 | Mesenchymal Stem Cell Therapy for Acute Respiratory Distress Syndrome in Coronavirus Infection: A Phase 2-3 Clinical Trial | Iran | Not specified  **Dose:** protocol 1, two doses of cells 100×10E6 (±10%) at Day 0 and Day 2. Protocol 2, the same plus two doses of EVs at Day 4 and Day 6  **Primary Outcome(s):** Adverse events assessment [Time Frame: From baseline to day 28], Blood oxygen saturation [Time Frame: From baseline to day 14] | 20 Control 20 Exp 1 20 Exp 2 |  |
| IRCT20200217046526N2 | Mesenchymal Stem Cell Therapy for Acute Respiratory Distress Syndrome in Coronavirus Infection: A Phase 2-3 Clinical Trial | Iran | Not specified  **Dose:** IV two doses of MSCs 100*10E6 (±10%), at Day 0 and Day 2 or two doses of MSCs 100*10E6 (±10%), at Day 0 and Day 2 plus two doses of extracellular vesicles (EVs) on Day 4 and Day 6  **Primary Outcome(s):** Adverse events, Blood oxygen saturation | 20 Control 40 Exp |  |
| IRCT20200413047063N1 | Placental Mesenchymal Stem cells for treatment of ARDS in Coronavirus infection, Phase 1 and 2 Clinical Trials | Iran | Placenta  **Dose:** 3 doses, no available information  **Primary Outcome(s):** Adverse events, Blood oxygen saturation | 10 Control 10 Exp |  |
| IRCT20200418047121N2 | Investigation the adipose and placenta-derived mesenchymal stem cells effect on the respiratory distress syndrome in patients with COVID-19: a pilot study | Iran | Adipose tissue  **Dose:** No information available  **Primary Outcome(s):** Biomarker expression, Blood oxygen saturation, CT scan | 3 Control 3 Exp |  |
| NCT04366271 | Phase II Clinical Trial to Explore the Efficacy of Allogeneic Mesenchymal Cells From Umbilical Cord Tissue in Patients With Severe Pulmonary Involvement by COVID-19 | Spain | Umbilical cord  **Dose:** 1 infusion of undifferentiated allogeneic cells, no information available  **Primary Outcome(s):** Mortality due to lung involvement due to SARS-CoV-2 virus infection at 28 days of treatment | 53 Control 53 Exp |  |
| NCT04390139 | Prospective, Double-blind, Randomized, Parallel, Placebo-controlled Pilot Clinical Trial for the Evaluation of the Efficacy and Safety of Two Doses of WJ-MSC in Patients With Acute Respiratory Distress Syndrome Secondary to Infection by COVID-19 | Spain | Wharton-Jelly  **Dose:** IV 1E106 cells at day 1 and 3  **Primary Outcome(s):** All-cause mortality at day 28 [Time Frame: Day 28]  weeks] | 15 Control 15 Exp |  |
| NCT04392778 | What is the Effect of Mesenchy, mal Stem Cell Therapy on Seriously Ill Patients With COVID 19 in Intensive Care? (Prospective Double Controlled Study) | Turkey | Not specified  **Dose:** IV 3*10E6 at day 0, 3 and 6  **Primary Outcome(s):** Clinical improvement [Time Frame: 3 months] | 15 Control 15 Exp |  |
| NCT04382547 | Treatment of COVID-19 Associated Pneumonia With Allogenic Pooled Olfactory Mucosa-derived Mesenchymal Stem Cells | Belarus | Olfactory-Mucosa  **Dose:** No information available  **Primary Outcome(s):** Number of cured patients [Time Frame: 3 weeks] | 20 Control 20 Exp |  |
| NCT04416139 | Mesenchymal Stem Cells for the Treatment of Severe Acute Respiratory Distress Syndrome Due to COVID-19. Pilot Study | Mexico | Umbilical cord  **Dose:** 1 * 10E6 in single administration  **Primary Outcome(s):** Clinical signs, Functional Respiratory changes: PaO2 / FiO2 ratio [Time Frame: Three weeks] | 5 Control 5 Exp |  |
| IRCT20200421047150N1 | Assessment of safety, efficacy and effective dose determination of human umbilical cord Whartonâ€™s jelly mesenchymal stem cell transplantation on treatment of COVID-19 (coronavirus) pneumonia and complications in humans | Iran | Wharton’s jelly  **Dose:** 0.5 or 2 * 10E6 cells / kg body weight on day 1, 3, and 6  **Primary Outcome(s):** Mortality. Timepoint: Up to 28 days after starting the study. | 45 Control 45 Exp |  |
| IRCT20160809029275N1 | Evolution of Allogenic Mesenchymal stem cell- derived Umbilical cord transplantation for ARDS patients infected with COVID19. | Iran | Umbilical cord  **Dose:** IV 1 * 10E6 cells at 1, 3, and 6 days  **Primary Outcome(s):** Biomarker expression, Blood oxygen saturation, CT scan | 10 Control 10 Exp |  |
| NCT04366323 | Phase I / II Clinical Trial, Multicenter, Randomized and Controlled, to Assess the Safety and Efficacy of Intravenous Administration of Allogeneic Adult Mesenchymal Stem Cells of Expanded Adipose Tissue in Patients With Severe Pneumonia Due to COVID-19 | Spain | Adipose tissue  **Dose:** 80 * 10E6 cells  **Primary Outcome(s):** Mortality [Time Frame: 28 days], Adverse Event Rate [Time Frame: 12 months] | 13 Control 13 Exp |  |
| ISRCTN33578935 | Rationale and investigational study for the treatment of COVID-19 with severe viral pneumonia with isolated, placental, mesenchymal stem cell exosomes | Germany | **Dose:** IV purified exosomes, XoGlo®, which are isolated, neonatal, mesenchymal stem cell-derived extracellular vesicles at a dose of 0.2 mg/kg body weight each in a total of 15ml on day 1 and day 3  **Primary Outcome(s):** adverse events, Blood oxygen saturation | 32 Control 32 Exp |  |
| NCT04399889 | Pilot Study of Safety and Efficacy of Cord Tissue Derived Mesenchymal Stromal Cells (hCT-MSC) in COVID-19 Related Acute Respiratory Distress Syndrome (ARDS) | USA | Umbilical cord  **Dose:** IV 1 * 10E6 cells/kg body weight (max dose 100 million cells)  **Primary Outcome(s):** Safety | 15 Control 15 Exp |  |
| NCT04389450 | A Randomized, Double-Blind, Placebo-Controlled, Multicenter, Parallel-Group Phase II Study to Evaluate the Efficacy and Safety of Intramuscular Injections of PLX PAD for the Treatment of Severe COVID-19 | USA | Placenta  **Dose:** (i) PLX-PAD interval high dose - PLX-PAD cells administered via 15 IM injections (1 mL each). Each subject will be treated twice, with an interval of 1 week between treatments, (ii) PLX-PAD low dose - LX-PAD 300, single administration, second administration of placebo after 1 week. (iii) PLX-PAD low dose  PLX-PAD 300, single administration, second administration of placebo after 1 week.  **Primary Outcome(s):** Number of ventilator free days [Time Frame: 28 days] | 70 Control 70 Exp |  |
| NCT04445454 | Mesenchymal Stromal Cell Therapy for Severe COVID-19 Infection | Belgium | Bone marrow  **Dose:** 3 infusions of (1.5)-3.0 *10E6/kg body weight (from the same donor) at 3-4 days interval  **Primary Outcome(s):** Safety and efficacy (unspecified) [Time Frame: Day 28] | 20 Exp |  |
| NCT04444271 | Prospective, Randomized Phase 2 Clinical Trial of Mesenchymal Stem Cells(MSCs) for the Treatment of Coronavirus Disease 2019(COVID-19) | Pakistan | Bone marrow  **Dose:** 2 *10E6 cells/kg MSCs on days 1 and 7  **Primary Outcome(s):** Mortality Time Frame: 30 days post intervention] | 10 Control 10 Exp |  |
| NCT04457609 | Application of Umbilical Cord Mesenchymal Stem Cells as Adjuvant Therapy for Critically-Ill COVID-19 Patients | Indonesia | Umbilical cord  **Dose:** 1*10E6 unit cells/kg body weight  **Primary Outcome(s):** Clinical improvement, Blood oxygen saturation [Time Frame: 15 days] | 20 Control 20 Exp |  |
| NCT04461925 | Treatment of Coronavirus COVID-19 Pneumonia (Pathogen SARS-CoV-2) With Cryopreserved Allogeneic Multipotent Mesenchymal Stem Cells of the Placenta and Umbilical Cord | Ukraine | Placenta  **Dose:** Cryopreserved allogeneic cells(1 * 10E6 cells/kg body weight) at 2-days intervals: Day 1, 4, and 7  **Primary Outcome(s):** Clinical improvement [Time Frame: At baseline, Day 1, Week 1, Week 2, Week 4, Week 8] | 15 Control 15 Exp |  |
| NCT04397796 | Phase 1b Randomized, Double-Blind, Placebo-Controlled Study Of The Safety Of Therapeutic Treatment With Immunomodulatory Mesenchymal Stem Cells In Adults With COVID-19 Infection Requiring Mechanical Ventilation | USA | Bone marrow  **Dose:** IV, no information available  **Primary Outcome(s):** Mortality, [Time Frame: 30 days] Number of ventilator-free days [Time Frame: 60 days] | 23 Control 23 Exp |  |
| NCT04466098 | Multi-center, Randomized, Placebo Controlled, Interventional Phase 2A Clinical Trial Evaluating the Safety and Potential Efficacy of Multiple Dosing of Mesenchymal Stromal Cells in Patients With Severe Acute Respiratory Syndrome Coronavirus 2 (SARS-Cov-2) | USA | Not specified  **Dose:** 300 * 10E6 cells  **Primary Outcome(s):** Safety [Time Frame: Within 6 hours of the start of the infusion] | 10 Control 20 Exp |  |
| ACTRN12620000840987 | Phase I trial on safety and tolerability of bone-marrow derived mesenchymal stromal cells (MSC) for deteriorating COVID-19 pneumonia | Australia | Bone marrow  **Dose:** 2 * 10E6 cells/kg body weight at day 0 and 3  **Primary Outcome(s):** Safety [Time Frame: Within 30 days of the start of the infusion], Blood oxygen saturation, biochemistry Day 3, 7, 14, 30 and 90 following study intervention] | 10 Exp |  |
| NCT04525378 | Mesenchymal Stromal Cell-based Therapy for COVID-19-associated Acute Respiratory Distress Syndrome: a Pilot Clinical Study | Brazil | Not specified  **Dose:** 3 doses 2.5, 5, or 10 *10E7 cells with repeat after 2 days (either at low or intermediate dose  **Primary Outcome(s):** Mortality | 10 Control 10 Exp |  |
| ACTRN12620000612910 | A pilot, open-label, randomised controlled clinical trial to investigate early efficacy of CYP-001 in adults admitted to intensive care with COVID-19 | Australia | Mesenchymo-Angioblast (CYP-001)  **Dose:** IV 2 *10E6 cells/kg body weight (up to a maximum of 200 million cells) on two occasions (Day 1 and Day 3)  **Primary Outcome(s):** Safety, Blood oxygen saturation [at day 7] | 12 Control 12 Exp |  |
| NCT04400032 | Cellular Immuno-Therapy for COVID-19 ARDS (CIRCA-19) the Vanguard Study | Canada | Bone marrow  **Dose:** IV on each of 3 consecutive days, (i) 5*10E6 cells/unit dose (cumulative dose: 75 million MSCs), (ii) 50 *10E6 cells/unit dose (cumulative dose: 150 million MSCs), (iii) up to 90 *10E6 cells/unit dose (cumulative dose: up to 270 million MSCs).  Primary Outcome(s): Safety [Time Frame: At time of infusion until one year post-infusion] | 3x3 Exp |  |
| IRCT20190717044241N2 | Cell therapy in patients with COVID-19 using mesenchymal stem cells | Iran | Not specified  **Dose:** IV 2*10E6 / kg body weight by intravenous injection three times  **Primary Outcome(s):** Mortality, Number of Participants with ventilator-free Days by Day 28 [Time Frame: Day 28] | 5 Exp |  |
| IRCT20140911019125N8 | Study the effect of intravenous injection of dental pulp mesenchymal stem cells in treatment of patients with COVID-19 pneumonia- A Phase 2&3 Clinical Trial | Iran | Dental pulp  **Dose:** IV 40 * 10E6 cells  **Primary Outcome(s):** Clinical improvement, CT scan at the beginning of the study and 1 day and 2 days, 4 days, 7 days and 14 days after the start of the study. | 50 Control and 50 Exp |  |
| NCT04361942 | Double Blind, Placebo-controlled, Phase II Trial to Evaluate Safety and Efficacy of Allogenic Mesenchymal Stromal Cells MSV_allo for Treatment of Acute Respiratory Failure in Patients With COVID-19 Pneumonia (COVID_MSV) | Spain | Not specified  **Dose:** allogenic, 1 *10E6 cells/Kg body weight  **Primary Outcome(s):** Mortality [Time Frame: 28 days], number of patients withdrawal of invasive mechanical ventilation [Time Frame: 0-7 days] | 12 Control 12 Exp |  |
| NCT04565665 | Emergency Use Pilot Study of Cord Blood Derived Mesenchymal Stem Cells for Treatment of COVID-19 Related Acute Respiratory Distress Syndrome | USA | Cord blood  **Dose:** IV over 1-2 hours on day 1. Patients may receive a second infusion of MSCs within 7 days after the first infusion per physician discretion. No information available on dosage.  **Primary Outcome(s):** Safety [Time Frame: At day 30 post MSC infusion], Mortality [Time Frame: At day 30 post MSC infusion | 35 Control 35 Exp |  |
| NCT04371393 | Mesenchymal Stromal Cells for the Treatment of Moderate to Severe COVID-19 Acute Respiratory Distress Syndrome | USA | Mesenchymal Stromal Cells  (Remestemcel-L)  **Dose:** 2*10E6 cells/kg of body weight IV plus standard of care, administered twice during the first week, with the second infusion at 4 days following the first infusion (± 1 day)  **Primary Outcome(s):** Number of all-cause mortality [Time Frame: 30 days] | 150 Control 150 Exp |  |
| NCT04611256 | Adjuvant Therapy With Mesenchymal Stem Cells in Patients Diagnosed With COVID-19 in Critical Condition | Mexico | Adipose tissue  **Dose:** Two IV infusion of 1*10E6 cells /kg body weight  **Primary Outcome(s):** Days to clinical improvement [Time Frame: up to 25 days], Blood oxygen saturation [at day 25] | 10 Control 10 Exp |  |
| NCT04615429 | Double-blind, Randomized, Controlled, Clinical Trial to Assess the Efficacy of Allogenic Mesenchymal Stromal Cells in Patients With Acute Respiratory Distress Syndrome Due to COVID-19 | Spain | Not specified  **Dose:** 1*10E6 cells /kg body weight  **Primary Outcome(s):** Blood oxygen saturation [Time Frame: 7 days] | 10 Control 10 Exp |  |

**Footnote:** The information to compile the table was obtained consulting the (1) EU Clinical Trials Register at [www.clinicaltrialsregister.eu/ctr-search/search](https://www.clinicaltrialsregister.eu/ctr-search/search), (2) Chinese Clinical Trial Registry at [www.chictr.org.cn/enindex.aspx](http://www.chictr.org.cn/enindex.aspx), (3) ClinicalTrials.gov database at <https://clinicaltrials.gov/>, (4) Iranian Registry of Clinical Trials at [www.irct.ir/](https://www.irct.ir/), (5) Japanese Registry of Clinical Trials at [www.clinicaltrials.jp/](https://www.clinicaltrials.jp/), (6) Australian Clinical Trials at [www.australianclinicaltrials.gov.au/](https://www.australianclinicaltrials.gov.au/), (7) SRCTN registry at [www.isrctn.com/](https://www.isrctn.com/), (8) Clinical Trial Registry – India at - <http://ctri.nic.in>, and (9) Registro Basileiro de Ensaios Clinicos at <https://ensaiosclinicos.gov.br/>.
